# National analysis of urinary cadmium concentration and kidney stone: Evidence from NHANES (2011–2020)

**DOI:** 10.3389/fpubh.2023.1146263

**Published:** 2023-03-15

**Authors:** Zhenyang Ye, Zaizhi Chen, Jinyang Luo, Lijing Xu, Dongping Fan, Jia Wang

**Affiliations:** ^1^Department of Urology, West China Xiamen Hospital of Sichuan University, Xiamen, China; ^2^Department of Urology, West China Hospital, Sichuan University, Chengdu, China; ^3^Department of Anesthesiology, The Third Hospital of Xiamen Affiliated to Fujian University of Traditional Chinese Medicine, Xiamen, China

**Keywords:** cadmium, kidney stone, NHANES, risk factor, environment

## Abstract

**Background:**

The association between urinary cadmium and kidney stone risk is inconsistent in previous studies, which needs further exploration. This study was performed to explore the association between urinary cadmium and kidney stone.

**Materials and methods:**

Data from the National Health and Nutrition Examination Survey (2011–2020) were included and further analyzed. Urinary cadmium was stratified into quartiles with quartile 1 (Q1: 0.025–0.104 μg/L) and quartile 4 (Q4: 0.435–7.581 μg/L). Further weighted logistic regression was adopted to evaluate the association between urinary cadmium and kidney stone. A subgroup analysis was used to verify the findings. The non-linear association was examined using the restricted cubic spline (RCS) regression.

**Results:**

A total of 9,056 adults aged 20 years and above were included in this study. In the fully adjusted model, an increased risk of kidney stones was identified for quartile 2 (OR = 1.40, 95% CI = 1.06–1.84, *P* < 0.05), quartile 3 (OR = 1.18, 95% CI = 0.88–1.59, *P* > 0.05), and quartile 4 (OR = 1.54, 95% CI = 1.10–2.06, *P* < 0.05). A similar association was found between continuous cadmium increase and OR of kidney stones in the fully adjusted model (OR = 1.13, 95% CI = 1.01–1.26, *P* < 0.05). The RCS also indicated a non-linear association between urinary cadmium concentration and kidney stone risk (*P* for non-linear < 0.001).

**Conclusion:**

In summary, cadmium exposure is identified as a risk factor for kidney stones in this study. Their non-linear association makes demands on early intervention for the cadmium-exposed population. Medical interventions for kidney stone prevention should take cadmium exposure into account.

## 1. Introduction

Kidney stone is a major condition affecting ~15% of the general population (1). The incidence of kidney stones increases with age and leads to more than 80,000 persons being hospitalized each year in the United Kingdom (2). The prevalence of nephrolithiasis still faces a rapid upward trend globally (3). It was estimated that the economic burden of kidney stones exceeded 2 billion dollars in the United States (4). Beyond the direct burden of renal stones, kidney impairment and urinary infection, triggered by kidney stones, still have serious adverse effects on patients' health. Therefore, identifying the factors associated with renal stones is vital for early intervention and may attenuate the predicament.

The risky role of obesity, metabolic diseases, genetics, and poor dietary patterns in forming renal stones has been well recognized (5, 6). Among the risk factors, heavy metal exposure such as cadmium has gradually attracted researchers' attention. It has been reported that heavy metal exposure can increase the risk of urinary diseases such as erectile dysfunction, kidney stone diseases, and prostate cancer (7–9). However, the role of cadmium in the risk of renal stones is less investigated.

Cadmium is a nephrotoxic metal, mainly accumulated in the liver and kidney. It can be excreted from urine. Some observational studies have preliminarily examined the association between cadmium and kidney stone. In the United States, Ferraro et al. (10) included 15,690 participants and found that women with urinary cadmium >1 μg/g had a 1.40-fold risk of renal stones. Contrary to their findings, Thomas et al. (11) reported that dietary cadmium was not associated with kidney stone risk in two large prospective cohorts in the general population. In addition, a cross-sectional study in China enrolled 1,293 participants and found that co-exposure to cadmium and lead was associated with higher risks of renal stones (12); however, this increased risk was not significant in participants with cadmium exposure only. The association between cadmium and kidney stone is inconsistent in previous studies and still needs further exploration.

Urinary cadmium reflects the total body burden of cadmium (13). In this study, we used data from the National Health and Nutrition Examination Survey (NHANES) to explore the association between urinary cadmium and renal stones. The non-linear association between urinary cadmium and renal stones was also examined, which may provide more evidence for clinical intervention.

## 2. Materials and methods

### 2.1. Study population

All data used in this current research were obtained from the National Health and Nutrition Examination Survey, implemented by the National Center for Health Statistics. To reflect the noninstitutionalized civilian population residing in the 50 states and the District of Columbia, NHANES was designed as a stratified, multistage probability sample through a complex statistical process to reflect all resident population information. The interview of NHANES covers demographic, socioeconomic, physiologic, and biochemical indexes as well as other health-related issues. Unique sampling weight was assigned to each participant, and multidimensional indexed such as interviewed weights (interviews in the home), mobile examination center (MEC) weights, or urinary metals should be carefully applied in different situations. Sample information and processing methods from NHANES for epidemiological and health-related research can be publicly achieved from the online website (https://www.cdc.gov/nchs/nhanes/index.htm). Written informed consent was obtained from each participant in NHANES, and the survey protocol was approved by the NCHS Research Ethics Review Board.

We aggregated 5-year cycles' (10 years) data from NHANES (2011–2020). Among the 54,716 participants in the total sample, a total of 45,660 samples were excluded due to the missing urinary cadmium measurement and kidney stones, and age below 20 years. Finally, 9,056 US adults were enrolled for analysis.

### 2.2. Urinary cadmium exposure assessment

Urinary cadmium sampling weights were applied to analyze these data properly. During NHANES 2011–2020, urine specimens were processed and stored at the Division of Laboratory Sciences, National Center for Environmental Health, and Centers for Disease Control and Prevention for analysis. Moreover, urinary cadmium was evaluated by the inductively coupled plasma-mass spectrometer (ICP-MS). Details of laboratory processing and quality assurance were available on the survey website (https://wwwn.cdc.gov/Nchs/Nhanes/2011-2012/). Lower limits of detection (LLOD) for each metal, as well as the number and percentage of samples below the LLOD were presented in the detection rate. In cases where the result was below the limit of detection, the value below the LLOD was imputed as the lowest LLOD value.

### 2.3. Ascertainment of kidney stone

In the present analysis, the identification of kidney stone cases was conducted through interviews and self-reported records during the NHANES 2011–2020 survey. Referring to the interview guidelines, cases of kidney stones were defined by a computer-assisted personal interviewing system by trained interviewers in the home as the following question “Have you ever had kidney stones?”. This classification method was also used in prior published studies.

### 2.4. Covariate determination

Relevant covariates were considered potential confounding factors in our analysis, including sociodemographic characteristics (age, gender, race/ethnicity, education, and poverty income ratio), lifestyle factors (body mass index [BMI], alcohol use, and smoking), and chronic disease conditions (diabetes mellitus [DM] and cardiovascular disease [CVD]). Age was recorded as continuous values. Gender was dichotomized into men and women. Education was categorized into three groups (below high school, high school, and college or above). Race/ethnicity was categorized as non-Hispanic white, non-Hispanic Black, Mexican American, other Hispanic, and other race. The family poverty-to-income ratio was divided into three groups (<1.5, 1.5–3.5, and ≥3.5). BMI was calculated as body weight in kilograms divided by meters squared and categorized as <20, 20–25, 25–30, and ≥30. Alcohol use was categorized into never, former, mild, moderate, and heavy. Smoking status was defined as never, former, and now. According to the previous literature, PE status was categorized into three levels (none, low, and moderate-to-vigorous) (14). Urinary creatinine was obtained as continuous variables (mg/dL).

### 2.5. Statistical analysis

The characteristics of participants were described across quartiles of urinary cadmium, and between-group differences were examined using the chi-square test for categorical variables and variance analysis for continuous variables. Logistic regression was used as the main analysis approach to examine the association between urinary cadmium and the odds risk ratio of kidney stone. The dependent variable was a history of kidney stones (yes or no). The independent variable was quartiles of urinary cadmium concentration.

Three models were built separately. First, the crude model included no covariate. Second, the regression model adjusted sex and age. Third, the full model further adjusted for race, education, alcohol drinking, smoking, family income poverty, diabetes, BMI, physical activity, cardiovascular disease, and urinary creatinine. In addition, continuous cadmium concentration was taken as an independent variable instead of the quartile of cadmium as sensitivity analysis. In addition, the median quartiles of cadmium were included in the earlier models to test the linear trend of association.

Based on the results of the logistic regression models, a significant relationship between urinary cadmium concentration and kidney stones was further examined. A dose–response relationship was estimated using a cubic spline, and three knots were established in our models. In addition, subgroup analyses were conducted to explore the influence of various characteristics on the association. All analyses were performed using R software (version 4.2.0), and *P* < 0.05 were regarded as statistically significant.

## 3. Results

### 3.1. Basic characteristics of the study participants

We included 9,056 adults aged 20 years or older in the present analyses. The prevalence of kidney stones was 10.82 %. The urinary cadmium concentration was categorized into quartiles (Q1: 0.025–0.104, Q2: 0.105–0.218, Q3: 0.219~0.435, and Q4: 0.435–7.581). The average age was 50.50 ± 17.32 years old. The participants in quartile 4 were more likely to be older, women, non-Hispanic white and Black, lower family income poverty, never smoking, and alcohol use, underweight, lower physical activity, with diabetes, with cardiovascular disease, and receive education below university (*P* < 0.001). Detailed baseline characteristics of this study are shown in Table 1.

**Table 1:** Characteristics of sample in NHANES during 2011 and 2020 (*n* = 9,056).

**Characteristics**		**Q1**	**Q2**	**Q3**	**Q4**	***P*-value**
		***N*** = **2,275**	***N*** = **2,254**	***N*** = **2,271**	***N*** = **2,256**	
Age (years)		42.5 (16.8)	48.8 (17.1)	52.9 (16.4)	57.1 (14.8)	<0.001
Education	Above high school	1,873 (82.3%)	1,842 (81.7%)	1,799 (79.2%)	1,627 (72.1%)	<0.001
	High school	197 (8.7%)	237 (10.5%)	256 (11.3%)	387 (17.2%)	
	Less than high school	205 (9.0%)	174 (7.7%)	213 (9.4%)	241 (10.7%)	
	Missing	0 (0.0%)	1 (0.0%)	3 (0.1%)	1 (0.0%)	
Gender	Male	1,143 (50.2%)	1,191 (52.8%)	1,126 (49.6%)	1,019 (45.2%)	<0.001
	Female	1,132 (49.8%)	1,063 (47.2%)	1,145 (50.4%)	1,237 (54.8%)	
Race	Non-Hispanic white	952 (41.8%)	808 (35.8%)	795 (35.0%)	737 (32.7%)	<0.001
	Other Race	342 (15.0%)	369 (16.4%)	404 (17.8%)	432 (19.1%)	
	Non-Hispanic Black	352 (15.5%)	469 (20.8%)	568 (25.0%)	667 (29.6%)	
	Mexican American	354 (15.6%)	362 (16.1%)	284 (12.5%)	226 (10.0%)	
	Other Hispanic	275 (12.1%)	246 (10.9%)	220 (9.7%)	194 (8.6%)	
Family income poverty	0–1.5	667 (29.3%)	662 (29.4%)	736 (32.4%)	834 (37.0%)	
	1.5–3.5	641 (28.2%)	659 (29.2%)	643 (28.3%)	671 (29.7%)	<0.001
	3.5-	740 (32.5%)	714 (31.7%)	655 (28.8%)	438 (19.4%)	
	Missing	227 (10.0%)	219 (9.7%)	237 (10.4%)	313 (13.9%)	
Alcohol drinking	Never	281 (12.4%)	278 (12.3%)	247 (10.9%)	240 (10.6%)	<0.001
	Former	134 (5.9%)	169 (7.5%)	195 (8.6%)	233 (10.3%)	
	Mild	729 (32.0%)	783 (34.7%)	741 (32.6%)	625 (27.7%)	
	Moderate	401 (17.6%)	296 (13.1%)	335 (14.8%)	316 (14.0%)	
	Heavy	462 (20.3%)	405 (18.0%)	345 (15.2%)	361 (16.0%)	
	Missing	268 (11.8%)	323 (14.3%)	408 (18.0%)	481 (21.3%)	
Smoke	Never	1,583 (69.6%)	1,466 (65.0%)	1,233 (54.3%)	863 (38.3%)	<0.001
	Now	269 (11.8%)	275 (12.2%)	420 (18.5%)	749 (33.2%)	
	Former	423 (18.6%)	513 (22.8%)	618 (27.2%)	642 (28.5%)	
	Missing	0 (0.0%)	0 (0.0%)	0 (0.0%)	2 (0.1%)	
BMI (kg/m^2^)	0–20	100 (4.4%)	69 (3.1%)	82 (3.6%)	135 (6.0%)	
	20–25	597 (26.2%)	484 (21.5%)	470 (20.7%)	537 (23.8%)	<0.001
	25–30	733 (32.2%)	722 (32.0%)	730 (32.1%)	699 (31.0%)	
	30-	822 (36.1%)	951 (42.2%)	958 (42.2%)	847 (37.5%)	
	Missing	23 (1.0%)	28 (1.2%)	31 (1.4%)	38 (1.7%)	
Physical activity	High	1,306 (57.4%)	1,210 (53.7%)	1,126 (49.6%)	957 (42.4%)	<0.001
	Low	283 (12.4%)	299 (13.3%)	312 (13.7%)	339 (15.0%)	
	Middle	216 (9.5%)	229 (10.2%)	256 (11.3%)	223 (9.9%)	
	Missing	470 (20.7%)	516 (22.9%)	577 (25.4%)	737 (32.7%)	
DM	No	1,860 (81.8%)	1,613 (71.6%)	1,507 (66.4%)	1,499 (66.4%)	<0.001
	Yes	386 (17.0%)	620 (27.5%)	736 (32.4%)	743 (32.9%)	
	Missing	29 (1.3%)	21 (0.9%)	28 (1.2%)	14 (0.6%)	
CVD	No	2,154 (94.7%)	2,044 (90.7%)	2,000 (88.1%)	1,873 (83.0%)	<0.001
	Yes	121 (5.3%)	210 (9.3%)	269 (11.8%)	383 (17.0%)	
	Missing	0 (0.0%)	0 (0.0%)	2 (0.1%)	0 (0.0%)	
Kidney stone	No	2,103 (92.4%)	2,011 (89.2%)	2,042 (89.9%)	2,021 (89.6%)	<0.001
	Yes	172 (7.6%)	243 (10.8%)	229 (10.1%)	235 (10.4%)	

Q, quartile; DM, diabetes; CVD, cardiovascular disease.

### 3.2. Associations between urinary cadmium and kidney stone

The associations between urinary cadmium and kidney stone are shown in Table 2. Based on quartile 1, a positive association between cadmium and kidney was identified at the Q4 level for all three models [Model 1: OR (95% CI) as 1.42 (1.16, 1.75); Model 2: OR (95% CI) as 1.04 (0.96, 1.11); and Model 3: OR (95% CI) as 1.13 (1.01, 1.26)]. A similar association was found between continuous Cd increase and odd risk of kidney stones in the full model (OR:1.13, 95% CI:1.01,1.26). In addition, the linear trend of elevated cadmium concentration and odds risk of kidney stone was identified (*P* for trend = 0.043).

**Table 2:** Logistic regression of kidney on quantile Cd levels.

**Model**	**Cadmium (**μ**g/L)**				**Continuous Cd increase (μg/L)**	***P* for trend**
	**Q1**	**Q2**	**Q3**	**Q4**		
Model 1	1.00	1.48 (1.20–1.81)	1.37 (1.11–1.69)	1.42 (1.16–1.75)	1.11 (1.04–1.19)	<0.001
Model 2	1.00	1.33 (1.08–1.64)	1.17 (0.95–1.45)	1.16 (0.94–1.44)	1.04 (0.96–1.11)	0.470
Model 3	1.00	1.40 (1.06–1.84)	1.18 (0.88–1.59)	1.51 (1.10–2.06)	1.13 (1.01–1.26)	0.043

Model 1 included no covariate.

Model 2 adjusted sex and age.

Model 3 adjusted sex, age, race, education, alcohol drinking, smoking, family income poverty, diabetes, BMI, physical activity, cardiovascular disease, and urinary creatinine.

### 3.3. Subgroup analysis

Subgroup analysis was conducted across various characteristics (Table 3). We did not find significant effect modification detected in age, sex, race, education, alcohol drinking, smoking, family income poverty, diabetes, cardiovascular disease, BMI, and physical activity (*P* > 0.05), although more substantial effects were observed in ages below 60 years, women, other Hispanic, less than high school, never alcohol drinking, never smoking, without diabetes and cardiovascular disease, overweight/obese, and low physical activity.

**Table 3:** Subgroup analysis of the association between Cd and kidney stone across characteristics.

**Characteristics**	**Cadmium levels (μg/L)**	***P* for interaction**
Age (years)		0.272
<60	1.23 (1.05–1.45)	
≥60	1.10 (0.95–1.28)	
Sex		0.296
Male	1.05 (0.91–1.22)	
Female	1.28 (1.09–1.50)	
Race		0.498
Non-Hispanic white	1.16 (1.00–1.36)	
Other Race	1.27 (0.92–1.75)	
Non-Hispanic Black	1.04 (0.79–1.37)	
Mexican American	0.94 (0.63–1.39)	
Other Hispanic	1.36 (0.95–1.94)	
Education		0.099
Above high school	1.18 (1.05–1.32)	
High school	0.67 (0.45–1.02)	
Less than high school	1.26 (0.70–2.28)	
Alcohol drinking		0.170
Never	1.95 (1.27–3.00)	
Former	0.99 (0.70–1.41)	
Mild	0.99 (0.83–1.18)	
Moderate	1.08 (0.86–1.37)	
Heavy	1.33 (1.03–1.70)	
Smoking		0.068
Former	0.90 (0.72–1.13)	
Never	1.25 (1.08–1.46)	
Now	1.21 (0.97–1.53)	
Family income poverty		0.990
0–1.5	1.17 (0.98–1.40)	
1.5–3.5	1.17 (0.95–1.44)	
3.5–	1.10 (0.91–1.33)	
DM		0.254
No	1.20 (1.06–1.37)	
Yes	1.03 (0.83–1.27)	
CVD		0.737
No	1.17 (1.04–1.31)	
Yes	0.91 (0.63–1.32)	
BMI		0.884
Underweight	1.10 (0.85–1.41)	
Normal	1.11 (0.94–1.30)	
Overweight/obese	1.22 (1.02–1.46)	
Physical activity (MET/week)		0.543
High	1.13 (0.99–1.29)	
Middle	1.03 (0.74–1.44)	
Low	1.27 (0.99–1.64)	

Logistic regression was conducted for increased Cd and kidney stone across characteristics. Characteristic variables were adjusted except for the subgroup variable.

### 3.4. Dose–response curve using cubic spine

**Figure 1:**
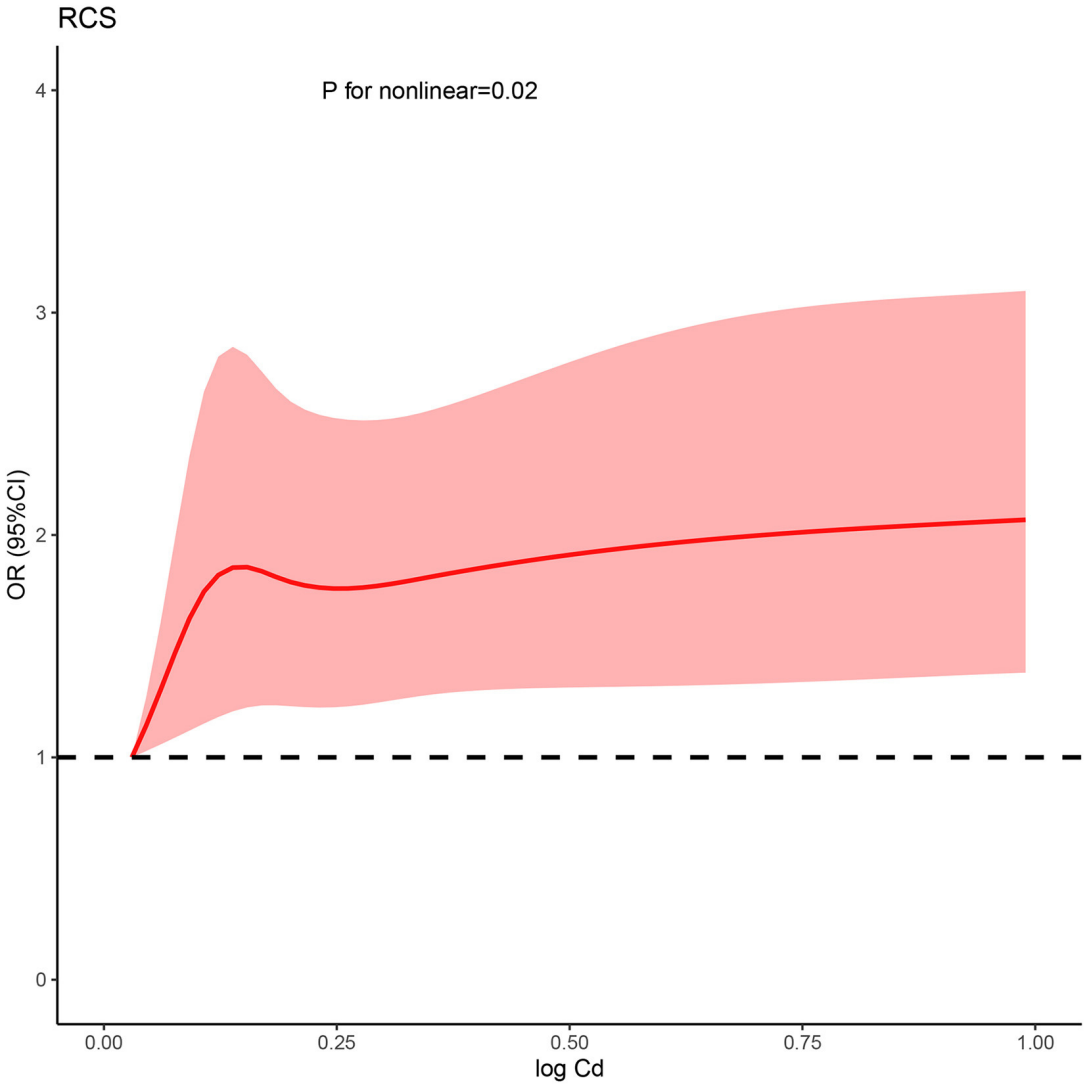
Non-linear relationship between Cd and kidney stone risk. RCS, restricted cubic spline; Cd, cadmium; OR, odds ratio; CI, confidence interval.

Based on the fully adjusted model, a restricted cubic spline was used to examine the dose–response relations between urinary cadmium concentration and kidney stone (Figure 1). It was found that urinary cadmium concentration was gradually associated with the odds risk of kidney stone, and a non-linear response was found, which suggested that a sharp increase was in early concentration exposure, and a stable curve was in later concentration exposure (*P* for non-linear was < 0.001).

## 4. Discussion

In this study, the increased risk of kidney stones is noted for participants with higher urinary cadmium. The risk is non-linear, with a sharp increase in early concentration exposure, and a stable curve in later concentration exposure. Therefore, intervention for cadmium exposure should be considered in the early stage.

Although some studies have explored the relationship between cadmium exposure and osteoporosis, there is limited national research on urinary cadmium and kidney stone, especially in the general population. In America, Ferraro et al. (10) reported that women with urinary cadmium of >1 μg/g creatinine had a higher risk of kidney stones. However, the non-linear association was not detected in their survey, which is addressed in our study. The non-linear association indicates that earlier intervention, instead of >1 μg/g, may provide more benefits. A similar significant association was also observed in two previous studies (15, 16). Of note, in a cohort study with an ample sample size (35,545 men and 33,050 women), no significant association was found between dietary cadmium intake and kidney stones (hazard ratio = 0.97 for men and 0.99 for women), contrary to our findings (11). However, it should be highlighted that dietary cadmium exposure may not reflect the actual cadmium burden in the body. In general, plasma cadmium principally reflects recent exposures to cadmium, and urinary cadmium indicates the total body burden of cadmium (13). Therefore, the inconsistent association may be explained by different definitions of cadmium exposure. In addition, Huang et al. (12) also reported that co-exposure to cadmium and lead could significantly increase the risk of kidney stones. Exposure to heavy metal is generally not isolated but coexistent. Therefore, the intervention for heavy metal exposure should be considered comprehensively.

In our study, the gender difference in kidney stone risk is identified. Women are found to have higher urinary cadmium than men. This discrepancy has been observed in some previous observational studies (10, 17). In general, due to higher estrogen levels, women tend to share a lower risk of kidney stones than men (18). The biological mechanism explaining the gender difference remains unknown. It was proposed by Ferraro et al. that absorption from the intestine through the divalent metal transporter-1 may be responsible for the gender difference. However, evidence from biological experiments is still lacking, which needs further verification in further studies (10).

The association between cadmium exposure and kidney stone has been established. However, the mechanism linking cadmium exposure to renal stone formation is not well elucidated. Several possible mechanisms may be responsible. First, cadmium is a nephrotoxic metal, leading to increased creatinine and kidney impairment (19). Accordingly, kidney impairment reduces the excretion of urine, followed by local mineral residues and then renal stone formation (20). The injury from cadmium may be attributed to a unified mechanism, oxidative damage (19). In addition, it was reported by Fuster et al. (21) that estrogen was positively associated with urinary calcium, indicating the risk role of sex steroid hormones in renal stone formation. Cadmium is an endocrine-disrupting chemical, which can mimic the function of estrogen (22). Therefore, the disrupted endocrine system may be an important mediator in promoting renal stone formation.

This study has some advantages and demerits. The major merit is the nationwide data from NHANES, providing ample sample size. The sampling weights are also considered in analyses, enhancing the power of statistical inference. Second, this study uses urinary cadmium to define cadmium exposure, which reflects the total body burden of cadmium. The participants are also collected from the general population, rather than from subjects with severe exposure to cadmium. However, there are also several limitations of this study. The main demerit is the cross-sectional design. The association should be interpreted with caution since the renal stone associated with kidney injury can also disrupt the excretion of cadmium. The causal association cannot be clarified in this study, requiring further investigation. In addition, we only investigate the exposure of cadmium rather than co-exposure with other heavy metals. The calculated effect size may be diminished. Moreover, the synergistic effects of co-exposure cannot be elucidated. Last but not least, some covariates interacting with cadmium and kidney function are not included in the current analyses, such as albuminuria and glomerular filtration rate, and protein intake.

In conclusion, cadmium exposure is identified as a risk factor for kidney stones in this study. Their non-linear association makes demands on early intervention for cadmium-exposed population. Medical interventions for kidney stone prevention should take cadmium exposure into account.

## 5. Conclusion

In summary, cadmium exposure is a risk factor for kidney stones. Early intervention may benefit the cadmium-exposed population. Medical interventions for kidney stone prevention should take cadmium exposure into account.

## Data availability statement

The datasets presented in this study can be found in online repositories. The names of the repository/repositories and accession number(s) can be found below: https://www.cdc.gov/nchs/nhanes.

## Author contributions

ZY, ZC, and JL: conceptualization. ZY, ZC, LX, and JW: data curation. ZY and DF: formal analysis. ZY and JW: writing the original draft, supervision, writing, reviewing, and editing. All authors contributed to the article and approved the submitted version.
